# Clowning as a supportive measure in paediatrics - a survey of clowns, parents and nursing staff

**DOI:** 10.1186/1471-2431-13-166

**Published:** 2013-10-10

**Authors:** Claus Barkmann, Anna-Katharina Siem, Nino Wessolowski, Michael Schulte-Markwort

**Affiliations:** 1 Forschungsgruppe Epidemiologie und Evaluation, Klinik für Kinder- und Jugendpsychiatrie, -psychotherapie und -psychosomatik (W29), Universitätsklinikum Hamburg-Eppendorf Martinistraße 52, Hamburg, D-20246, Germany

**Keywords:** Humour, Hospital clowning, Paediatrics, Hospital treatment, Programme evaluation

## Abstract

**Background:**

Hospital clowns, also known as clown doctors, can help paediatric patients with the stress of a hospitalization and to circumvent the accompanying feelings of fear, helplessness and sadness, thus supporting the healing process. The objectives of the present study were to clarify the structural and procedural conditions of paediatric clowning in Germany and to document the evaluations of hospital clowns, parents and hospital staff.

**Methods:**

A nationwide online survey of hospital clowns currently active in paediatric departments and an accompanying field evaluation in Hamburg hospitals with surveys of parents and hospital staff were conducted. In addition to items developed specifically for the study regarding general conditions, procedures, assessments of effects and attitudes, the Work Satisfaction Scale was used. The sample included n = 87 hospital clowns, 37 parents and 43 hospital staff members.

**Results:**

The online survey showed that the hospital clowns are well-trained, motivated and generally satisfied with their work. By their own estimate, they primarily boost morale and promote imagination in the patients. However, hospital clowns also desire better interdisciplinary collaboration and financial security as well as more recognition of their work. The Hamburg field study confirmed the positive results of the clown survey. According to the data, a clown intervention boosts morale and reduces stress in the patients. Moreover, there are practically no side effects. Both parents and hospital staff stated that the patients as well as they themselves benefited from the intervention.

**Conclusions:**

The results match those of previous studies and give a very positive picture of hospital clowning, so that its routine use and expansion thereof can be recommended. Furthermore, the intervention should be subject to the rules of evidence-based medicine like other medical treatments.

## Background

Children and adolescents who are severely physically ill and require hospitalisation represent a special responsibility for the health care system and the people working in it for a number of reasons: In addition to the illness itself, the young patients are also stressed by the separation from their parents, the strange environment, the fear of painful treatments and/or the uncertainty of the treatment outcome e.g. [1,2]. Hospital clowns, also known as clown doctors, can help paediatric patients with these stressors and to circumvent the accompanying feelings of fear, helplessness and sadness, thus supporting the healing process. They can be friends in need who help to bear a difficult situation more easily or simply offer a welcome distraction from the ward routine e.g. [3,4].

Hospital clowning was developed in 1986 in the United States by Michael Christensen, a co-founder of the New York-based Big Apple Circus, and spread quickly from there throughout Europe. The first German association of clown doctors was founded in 1994 by a student of Christensen’s named Laura Fernandez. In the meantime, according to estimates of the umbrella organisation “Clowns für Kinder im Krankenhaus Deutschland e. V.” [Clowns for children in hospitals Germany], founded in 2004, there are roughly 250 hospital clowns working around the country. Most of them are members of one of the approximately 40 regional associations and work not only in hospitals but in dialysis centres, children’s hospices or paediatric emergency rooms. The specific work of a clown can be described well by three different role models: (1) the entertainer uses the performing arts, e.g. magic/sleight of hand, (2) the auguste, who makes children laugh using humour and slapstick acts, and (3) the clown as an ally who offers the child emotional support [5].

The mode of action of hospital clowning can be specified at four different levels of impact (see also [3,6-10]). At the physiological level, laughing stimulates and modulates the immune system via the release of endorphins. At the emotional level, laughing replaces negative feelings with positive ones. At the cognitive level, the performance distracts the patient from his own situation, fosters imagination and supports the learning of new ways to express oneself. At the social level, laughing creates a connection between the children and clowns and stimulates further interaction. For each of the four levels of impact, there are an entire series of independent studies in which specific aspects were empirically tested and the corresponding hypotheses regarding the modes of action were confirmed (e.g. [11], on laughing and muscle tension; [12], on laughing and breathing; [13], on humour and fear, [14], on laughing and immunoglobulin A; [15], on laughing and pain).

In addition to the aforementioned primary effects, side effects can also occur. For example, the offer of a clinic clown in a hospital has the effect of creating publicity for the relevant institution. For the parents of paediatric patients and the hospital staff, the clown’s visits likewise provide distraction, stress relief and support. However, if a clown does not comply with the rules of a hospital or crosses the personal boundaries of a patient, there may be negative side effects. These include, for example, wasting money, disruption of workflows, annoyance and irritation among hospital staff and parents as well as helplessness, overtaxing and discontent among patients.

A search in the relevant abstract databases Embase, Medline and PsycINFO revealed a multitude of comments and case reports in predominantly low ranked journals. However, there exist several sample studies fulfilling more rigorous scientific criteria. These can be divided thematically into two groups, namely, controlled trials and evaluations of effectiveness under routine conditions. Systematic reviews or meta analyses do not exist to date.

Currently, there are a total of nine randomized controlled trials on the effect of hospital clowning during specific medical interventions. Five of these investigations deal with the presence of hospital clowns before, during, and/or after surgery or anesthesia induction and the possible reduction of anxiety [16-20]. One study analyzes the effect of clown intervention during botulinum toxin injections [21]. Three trials [22-24] investigated a possible long-term effect (up to one day later) in the context of an in-patient hospital stay. Overall, the fear-and stress-reducing effect of hospital clowning could be detected in most studies, but not in all. In particular, a long-term effect beyond the actual duration of the clown visit has not been identified conclusively.

With regard to the attitudes and subjective assessments of the impact by the patients, parents and hospital staff that are of interest in the context of the present study, four independent studies have been published up to now:

– Loidl-Keil et al. [25] evaluated the performances of hospital clowns in three different hospitals in Upper Austria. They surveyed n = 37 patients (3–20 years old, M = 11 year) and n = 98 nurses using a questionnaire on the acceptance and subjective experience of efficacy of the clown visits. The results of the evaluation were very positive overall. The clear majority of the children enjoyed the clown performances, wanted more frequent clown visits and preferred these to other entertainment activities on the ward. Only a small number of respondents reported feeling unwell or ill at ease or disturbed. The nursing staff gave comparable assessments.

– Battrick et al. [26] evaluated the clown visits in an English hospital using a questionnaire from the point of view of n = 49 children, 43 parents, 93 hospital staff members and 17 physicians. The results showed a very positive reception by all groups. The vast majority (82%) of the children enjoyed the clown performances. Only 3 (6%) stated that they didn’t like the clowns. Almost all of the parents and hospital staff claimed that the presence of a clown doctor had a positive influence on sick children and their families. The physicians made comparable statements.

– Glasper et al. [27,28] studied the topic in three different study modules at an English children’s hospital. The result of two focus groups with n = 5 and 7 clowns showed that hospital clowns are well trained and take their profession very seriously. They believe they can improve the children’s care and perceive themselves as valued members of the hospital team. However, problems with scheduling appointments and prejudices of hospital staff were also mentioned. The survey of various groups of people (n = 17 physicians, 93 nursing staff, 43 parents and 49 children) confirmed the assessments of the focus group. Nearly all of the respondents valued the work of the clowns and believed that the performances would have a positive influence on the health of the patients. However, a few of the physicians stated that they personally did not like hospital clowns. In the third study module, n = 20 patients between the ages of 4 and 11 were asked to show in drawings and stories how they felt about a hospital stay before and after a clown visit. Before the clown visit, mostly negative comments (28 of 35) such as “sad”, “nervous” or “worried” were made, but after the clown visit, a significantly more positive perception appeared (57 positive and 3 negative comments).

– Koller and Gryski [3] surveyed n = 143 staff members and 51 parents regarding the clown visits in a paediatric clinic in Toronto. The vast majority (85%) of the staff had no concerns with respect to the clown visits. Just under half stated that they experienced the clowns as a support for their own work and almost all of them evaluated the clown program as beneficial to the hospital. The parents expressed equally positive opinions: more than three quarters reported that their children and they themselves enjoyed the clown visits and almost all parents considered their children to be happier after the performances than before (94%).

Previous results regarding efficacy under routine conditions show that clown doctors do valuable work and are appreciated by patients as well as parents and hospital staff. However, up to now there have been almost no such systematic, empirical, scientific studies in Germany on the use, effect and side effects of hospital clowning for hospitalised paediatric patients under routine conditions comprising more than one hospital. The present report describes an initial evaluation of the actual state of care provision in this field.

The initial questions of the present study were:

1. What is the care provision situation with regard to hospital clowning in Germany?

2. How do hospital clowns, parents and ward staff rate the intervention?

## Method

### Design

Each question was processed using a separate sub-study. There were no existing registers, archives or other form of documentation that could be used to answer the first study question. Thus, the care provision data were collected directly from the hospital clowns via a nationwide online survey. How a clown performance is actually judged was determined in an exemplary manner in Hamburg hospitals via a multi-site cross-sectional survey. The hospital clowns were observed during their work and the parents and hospital staff were surveyed using standardised questionnaires.

### Variables and instruments

The dimensions and variables to be investigated were selected on the basis of previous studies e. g. [12,13,17,22] and mostly study-specifically operationalised. All questionnaires contained primarily completely standardised items that were identically worded as far as possible with a five-point response scale, along with a small portion of free-response questions. The seven-page online questionnaire for hospital clowns covered the seven dimensions listed below with up to 25 items each (91 items in total, see Additional file 1).

1. General information about working as a clown (working hours, hourly wage, etc.)

2. General conditions for the performances in paediatric clinics (number, frequency, etc.)

3. Procedure for the performances in paediatric clinics (duration, elements, etc.)

4. Patients, parents and hospital staff (effect and side effects)

5. Work satisfaction (10-item short form of the Work Satisfaction Scale [ABZ], [29]

6. Miscellaneous (image, demand, etc., not reported here)

7. Personal information (sociodemography)

For the field evaluation, separate two-page questionnaires were developed for parents and hospital staff (see Additional files 2 and 3). The questionnaire for parents covered personal data, general information about the hospital stay and the assessment of the effect of the intervention on the patients. In addition, the parents were able to express their attitude towards hospital clowning and make suggestions for improvement. The questionnaire for hospital staff likewise covered personal data, the effect of the performances on patients, their attitudes towards hospital clowning and possible improvements. The study was exempt from ethical approval because no experiments were performed and no patient data was collected. Clowns, parents and hospital stuff were informed and gave their written consent according to the declaration of Helsinki.

### Samples

#### Nationwide online survey

The target population for the online survey consisted of all hospital clowns currently working in paediatric clinics in Germany. The sample was drawn from the corresponding address register of the umbrella organisation of hospital clowns (see Background). In addition, all regional associations were asked to solicit participation by the clowns known to them. The actual establishment of contact and recruitment were done via e-mail. Of the roughly 250 hospital clowns currently active nationwide according to information provided by the umbrella organisation, n = 141 (56%) took part in the survey between June and September 2011. However, n = 54 respondents (38.3%) had to be excluded because they only worked abroad, only worked with older patients, or did not complete the questionnaire (n = 2, 2 and 50). The resulting sample for analysis consisted of n = 87 analysable questionnaires with an overhang of hospital clowns from Bavaria (22.8%). This overhang can be explained by the fact that the umbrella association there provided a great deal of support for the survey. However, the predictive analyses (see section 3.1.4) showed that this did not lead to a bias of the assessments. The most important characteristics of the hospital clowns surveyed are summarised in Table 1.

**Table 1:** Characteristics of the clown sample

**Variable**	**Label**	**%**
Sex	female	70.1
Native country	Germany	88.5
Marital status	single	23.0
	married, partnership	60.9
	divorced, separated, widowed	16.1
Own children	yes	58.8
Education	secondary school	14.9
	high school	31.0
	university	51.7
	other	2.3
Job training	acting	17.4
(% of statements)	health care	16.5
	paedagogy	15.7
	clowning	14.9
	arts, culture, music	11.6
	student	3.3
	nature science	2.5
	other	18.2

note. n = 87 hospital clowns.

#### Hamburg hospital survey

The field survey of parents and hospital staff during the intervention was carried out in collaboration with the Hamburg association of hospital clowns (Klinikclowns Hamburg e. V.). Accordingly, the intervention sample consisted of the hospital clown performances in Hamburg during the period of the study. The sample covered eight hospital clowns with a total of seven performances on paediatric surgery, oncology and orthopaedics wards in four different hospitals (Asklepios Klinik Nord, Kinderkrankenhaus Altona [Altona Children’s Hospital], Kinderkrankenhaus Wilhelmstift [Wilhelmstift Children’s Hospital], Universitätsklinikum Hamburg-Eppendorf [Hamburg-Eppendorf University Hospital]. All parents who were present at one of the performances and willing and able to fill out the questionnaire were included. The same applied to the hospital staff. This data collection likewise took place between June and September 2011. Of the parents present at the performances, 70% took part in the survey, while 60% of the hospital staff in attendance participated. The resulting sample included n = 37 parents and n =43 nursing staff members. The mothers provided 83.8% of the parental evaluations. The average age of the children was M = 7.1 years (SD = 4.64); 56.8% were male. The duration of hospitalisation ranged from 1 to 71 days (M = 8.7, SD = 15.16). For most of the children, this was the first clown visit during the current hospital stay (71.4%), the second for 20.0% and the third for 5.7%. The sample of hospital staff consisted primarily of nursing staff (79.1%; 9.3% were student nurses, 4 others).

### Analyses

Each part of the study was analysed separately. In addition to univariate and bivariate descriptive statistics, multiple linear regressions were used for modelling the estimated efficacy and work satisfaction of the hospital clowns. The analyses were carried out with PASW Statistics 18.0.

## Results

### Nationwide online survey

#### General conditions and procedure for the performances

The respondents had been working for a mean period of M = 7.4 years as hospital clowns (SD = 4.57). Most of them are members of a clown association (88.5%) and learned their job via seminars (35.4%), followed by job shadowing (27.2%), clown schools (24.7%), and other (12.7%). The vast majority are self-employed and work on a fee basis (83.3% of respondents), 15.6% are volunteers and 1.1% are salaried employees. The average hourly wage is M = 43.0 euros (SD = 12.71). The payor is usually a clown association (67.6% of responses), followed by hospitals (14.7%) and foundations (3.9%). Hospital clowns receive their payment from parents’ associations, special support groups or donations (13.7%).

The general conditions for the performances are summarised in Table 2. Slightly more than half of all patients (58.0%) are visited only once, 18.7% are visited 2–4 times, 8.3% 5–10 times and 15.2% more than 10 times. Half of the clowns also perform for patient groups (54.0%). The age of the patients is generally between M = 2.0 and 15.1 years (SD = 1.46 and 2.49). The institutions where the clowns perform are usually visited once a week (47.4%); (24.2% are visited once every fourteen days, 8.4% are visited once a month, 8.4% are visited twice a week and 11.6% are visited at individually determined intervals).

**Table 2:** General characteristics of performances

	**M**	**SD**	**Med**	**IQR**	**Min**	**Max**
Working hours per week	4.6	2.89	4.0	3.0	1	16
Patients per week	39.8	32.94	30.0	30.0	4	200
Lenght of performance in min	9.6	4.99	10.0	4.5	3	30
Group performances per week	1.4	1.04	1.0	1.0	1	6
Patients per group	7.2	4.63	6.0	4.0	2	20
Minimum age of patients in years	0.7	0.75	0.5	1.0	0	3
Number of hospitals	3.4	2.73	3.0	3.0	1	12

note. n = 87 hospital clowns.

For 35.6% of the hospital clowns, a doctor’s white coat is part of their costume. Every tenth clown always performs alone, i.e. without colleagues (10.8%). Asked about the main roles they play during their performances, the respondents stated that they play the auguste role 35.9% of the time, the role of an entertainer (with music, magic tricks, etc.) 23.0% of the time, the role of a whiteface clown 21.6% of the time and as a friend/health visitor for the child 19.5% of the time. The main elements of the performance were listed as music (34.7%), magic tricks (13.3%), pantomime (13.1%) and acrobatics (5.9%). In addition, there is a large group of other elements such as improvisation, slapstick or dance (32.9%).

On average, M = 5.0% of the children (SD = 3.31) who actually belong to the target group refuse to participate. The refusal is mostly given by the patients themselves (40.3% of responses), but also by parents (35.2%), nursing staff (20.4%) and physicians (4.1%). The most common reason is the need for rest or sleep (32.5%), but other reasons include fear (21.1%), lack of willingness (20.7%), pain (13.1%), lack of time (5.9%) and others (5.9%, primarily that the patients think they are too old, parents do not give consent, risk of infection).

#### Perceived effect on patients

**Figure 1:**
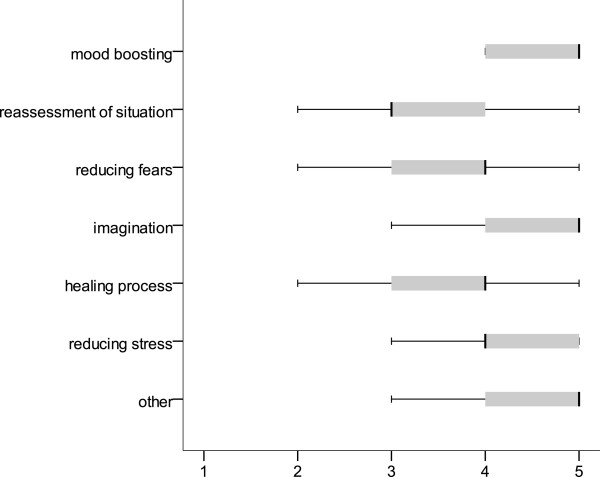
Effect of hospital clowning on patients from the clown’s perspective (1 = not at all, 2 = slight, 3 = medium, 4 = quite, 5 = very; n = 87).

Figure 1 summarises the processes in patients that hospital clowns hope to stimulate. These include primarily brightening the patients’ mood and stimulating their imagination. The “Other” category was likewise rated as very supportive, including self-confidence, self-perception and joy in life. A five-point rating scale was used to determine the degree of appreciation from different groups felt by the clowns in their work (1 = none at all, 5 = greatly appreciated). Based on this, the clowns reported that patients show the greatest appreciation, followed by parents and nursing staff (M = 4.8, SD = 0.39; M = 4.7, SD = 0.47; M = 4.1, SD = 0.69). Physicians show the least appreciation, even though the mean is still “quite appreciative” (M = 3.8, SD = 0.94).

#### Work satisfaction

**Figure 2:**
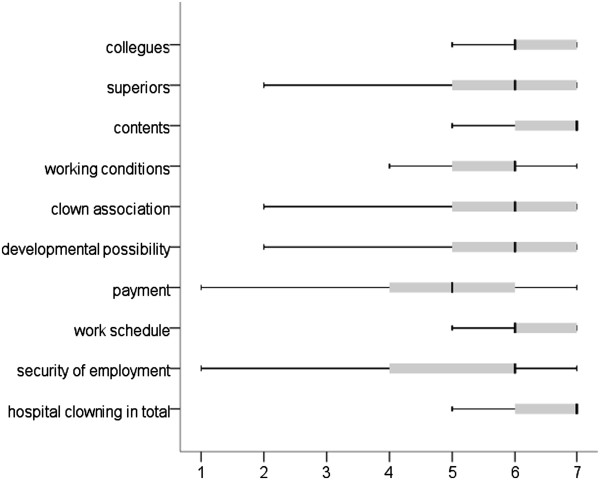
Work satisfaction of hospital clowns (1 = very dissatisfied, 7 = very satisfied; n = 87).

The evaluation of work satisfaction across the nine different factors as well as overall is summarised in Figure 2. According to this, the hospital clowns are very satisfied with their work on the whole. This applies primarily to the content of their work, but also to their colleagues. The least satisfaction was shown with regard to payment, but here as well the average rating was positive. In response to the question of particular sources of dissatisfaction, 20.1% of the hospital clowns gave 22 different answers, which could be summarised as follows (with multiple answers possible): 38.9% low level of appreciation, 27.8% low level of interdisciplinary contact and 16.7% low level of financial support. In addition, 38.9% of the responses could not be summarised (e.g. external intervention in the clown’s work, lack of staff in hospitals, competition among the associations themselves).

#### Prediction of the perceived effectiveness and work satisfaction

To operationalise the suspected effect of the hospital clowns in an outcome variable, the scalability of the corresponding six items from section 3.1.2 (excluding “Other”) was checked using exploratory factor analysis (principal component method, scree test). The correlation matrix at KMO = .66 was not suitable for this approach, but the scree test indicated a clear general factor model with 40.5% explained variance. The loadings of the items on this general factor were between a = .58 and .75; the reliability of the resulting scale was adequate for group statistics with Cronbach’s α = .70. However, exploratory bivariate analyses showed only a single, small effect with this construct; according to this, female hospital clowns rated the effect of their performance somewhat higher than male clowns did (r = .23, p = .033). Neither general, formal, nor particular content characteristics of the work or other personal characteristics of the clowns, such as professional experience, played a role.

To operationalise the work satisfaction of the hospital clowns as an outcome variable, the scalability of the nine satisfaction items from the previous section was reviewed (using the method described above). A scale with Cronbach’s α = .71 was identified using a general factor model (KMO = .72, 30.7% explained variance, loadings between a = .33 and .68). However, exploratory bivariate analyses showed that work satisfaction correlated with only two other variables:

– The appreciation experienced from the patients, their parents and the nursing staff (hardly any from the physicians) showed minor correlations with work satisfaction (r = .23, p = .035; r = .29, p = .007; r = .22, p = .039; r = .17, p = .107). According to this, the more appreciation the hospital clowns receive for their work, the greater their work satisfaction.

– When hospital clowns believe that the patients continue to benefit from the experience after the performance as well, their level of work satisfaction is likewise higher (r = .39, p = .000).

In a joint regression model with the predictors “appreciation from parents”, “appreciation from patients” and “patients continue to benefit after the performance”, a maximum explained variance of 20.0% is achieved for work satisfaction (F = 6.68, df = 3/83, p = .000; ß = .14, .11 and .33). This means that the job satisfaction is substantially associated with the self-perceived appreciation by parents and patients, as well as the sustainability of the effect on the patient.

### Hamburg hospital survey

#### Parental ratings

**Figure 3:**
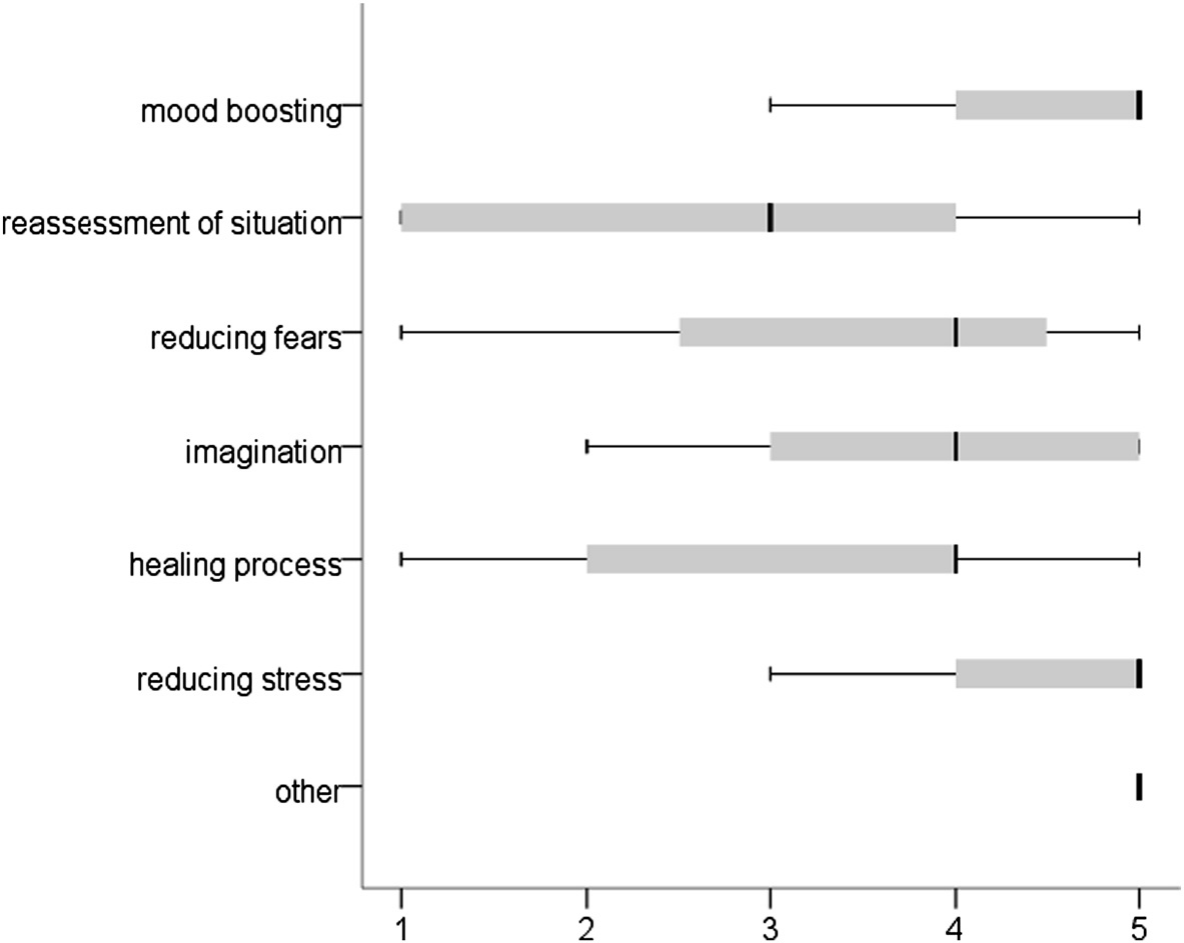
Effect of hospital clowning on patients from the parent’s perspective (1 = not at all, 2 = slight, 3 = medium, 4 = quite, 5 = very; n = 37).

On a five-point scale of 1 (not good at all) to 5 (very good), the surveyed parents stated that they could, on average, assess the effect of hospital clowning quite well (M = 4.3, SD = 0.86, min = 2, max = 5). Figure 3 shows how the parents surveyed rated the effect of hospital clowning on their children. According to this, a clown visit primarily boosts morale and reduces stress. In contrast, the influence on the patients’ re-evaluation of the situation was rated the lowest. In the “other” category, distraction was most frequently named.

Furthermore, the parents were asked to rate their level of agreement to different statements regarding hospital clowning (rating scale as in Figure 3). According to this, the vast majority of the children experienced no fear/anxiety at all before the clown visit (81.1%) and would be quite or very happy about additional performances (all together 91.9%). Every third parent stated that the presence of a clown during specific interventions (e.g. blood sampling) would be “very” or “quite” helpful to their child. Overall, two thirds of the parents were very satisfied with the clown visits (67.6%, 21.6% quite satisfied, 10.8% moderately satisfied). The majority of the parents (66.7%) felt that hospital clowns should perform 1–2 times per week; every fifth parent even thought a daily performance would be a good idea (19.4%). In response to the question of whether there was something the clowns could do differently or better, 13.5% answered “yes”; three respondents desired longer clown visits and two wanted a new programme.

#### Hospital staff ratings

**Figure 4:**
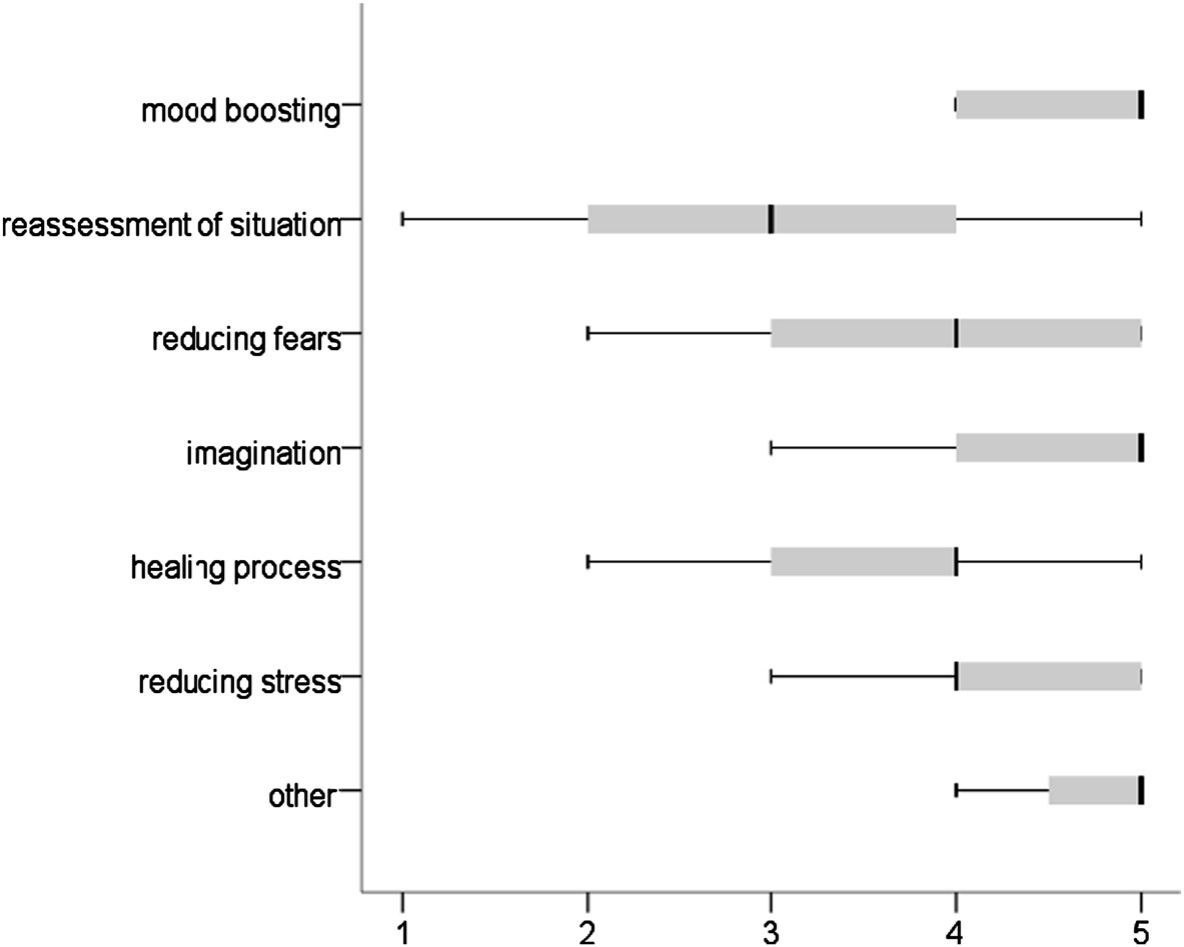
Effect of hospital clowning on patients from the hospital staff’s perspective (1 = not at all, 2 = slight, 3 = medium, 4 = quite, 5 = very; n = 43).

On a five-point scale of 1 (not good at all) to 5 (very good), most of the respondents stated that they could assess the effect of hospital clowning quite well (M = 4.2, SD = 0.65, min = 3, max = 5). Figure 4 shows the ratings of the hospital staff with regard to the effect of clown visits on patients. According to this, for paediatric patients a clown visit primarily boosts morale, reduces stress and stimulates their imagination. In the “other” category, distraction, an improvement in self-esteem and fun were most frequently named.

Furthermore, the hospital staff were asked to rate their level of agreement to different statements regarding hospital clowning (rating scale as in Figure 3). Overall, the responses present a positive picture: more than three quarters of the respondents would like more clown visits on the ward (78.6%) and nearly all of them viewed the performances as an enrichment of the daily routine on the ward (90.7%). Critical undertones, such as subtle criticism of the everyday ward routine or competition with the nursing staff, were found only in individual cases. However, hospital clowns are rarely asked to perform during specific interventions such as blood sampling (81.4% not at all). The only suggestion for improvement made was that the clown visits be more frequent (14%).

## Discussion

### Nationwide survey

The information about the general conditions convey a clear picture of the actual structure of the clown visits. Most clowns stated that they had undergone extensive training and regularly attended advanced training courses. Because membership in a clown association frequently requires regular participation in advanced training courses (the umbrella organisation requires at least one year of coaching), there is a functioning quality control mechanism. With the documentation of the hourly wage, previously non-existent transparency has been achieved, and with average rate of € 43.00 per hour, the clowns have been given a yardstick with which to assess their individual earnings. It is noteworthy that every seventh clown is a volunteer working without pay, which speaks for the high personal commitment of the clowns. Unfortunately, there is no direct and reliable standard of comparison available from similar professional groups.

The majority of the hospital clowns stated that they usually perform in a duo. The result can be explained by the fact that performing as a duo is a “desirable” acceptance criterion of the umbrella organisation. This is also described in the literature as being the most useful procedure [3,30], e.g. to relieve children of the pressure of active participation. Elements of the performances named, in addition to music and magic tricks, included pantomime, acrobatics, improvisation, slapstick and dance. The hospital clowns thus have a broad repertoire and must therefore document good training with regard to these skills. Based on the information collected, the low rejection rate can be explained by the generally high acceptance of the clown performances. However, other factors such as a preselection by the nursing staff or the strategic avoidance of potential rejection are also conceivable.

According to the assessments of the clown doctors, the clown visits had predominantly positive effects on patients, parents and hospital staff, especially with regard to mood. This assessment matches the evaluations of the parents and the hospital staff from the Hamburg hospital survey on the one hand and the results of other studies on the other (see introduction). In agreement with this, from the point of view of the hospital clowns, their work is greatly appreciated. They receive in relative terms the least appreciation from physicians, but this value is still on the positive half of the rating scale. This assessment on the part of the physicians is probably related to the rather sporadic contact between the two groups.

The hospital clowns are very satisfied with their work on the whole. Relative dissatisfaction and thus potential for improvement in the work situation exist with regard to recognition of their work, interdisciplinary contact and financial support. While the first two aspects could be resolved via a reorganisation of the collaboration of the professional groups involved, an improvement in pay can hardly be expected in financially difficult times. Comparable information about the satisfaction of hospital clowns or comparable professional groups are unfortunately not available.

As the correlation analyses show, the suspected effect on patients from the standpoint of the clowns can hardly be explained by any of the data collected. There is a small effect only for gender, indicating that female clowns rate their effect more highly than male clowns do. However, this effect should be interpreted with caution because of multiple testing. It is not possible to draw any conclusions in this study as to whether this is an effect of self-evaluation or corresponds to reality. The low predictive value of the variables involved may be a consequence of a reduced variance due to the effect of social desirability. In contrast, the work satisfaction can be somewhat better explained. The two predictors “experienced appreciation” and “experienced sustainability” are directly plausible and together are responsible for 20% of the effect variance.

### Hamburg hospital survey

In the Hamburg hospital survey, the participating parents stated that they could assess the effect quite well. The ratings of the parents confirm the assessments of the hospital clowns in the nationwide survey, according to which hospital clowning first and foremost boosted morale and relieved stress. Negative effects of the clown visits are hardly perceived at all. As mentioned above, these results match those of previous studies (see introduction). Visits from hospital clowns are thus a good intervention to improve patients’ moods at least in the short term. Parents also stated that most of the children had “no fear at all” during the first clown visit. This confirms the low rate of rejection stated by the clowns in the online survey and matches the controlled studies on fear reduction via hospital clowning. Thus, coulrophobia, the fear of clowns sometimes mentioned in this context, is generally not an issue. The actual rejections expressed were very few in number and were based on various reasons of a more practical nature. The statements regarding parental satisfaction confirmed the assessments of efficacy.

According to the assessments of the nursing staff, a clown visit has a uniformly positive influence on paediatric patients. Procedural disruptions or stress caused by the hospital clown occur only in individual cases. Overall, the hospital staff in the present study thus rated the work of the hospital clowns as very positive. As a result, more than two thirds of the respondents would like more hospital clown visits. Almost exclusively positive opinions of the hospital staff are found in the literature as well [10,12,13,17]. There is only one study [19], in which the medical staff primarily rejects clown visits, as they would disrupt the workflow. Thus, clown performances generally appear to be very compatible with ward routine. However, in the Hamburg hospitals, more than two thirds of the surveyed staff replied to the question of whether they called hospital clowns for specific interventions with “not at all”. Yet, the effect of fear reduction before surgical interventions was documented in several controlled studies (see also current state of research). The results thus also show that the hospital clowns are not yet fully integrated in the ward routine and that the potentials of this intervention have not yet been fully utilised.

## Conclusions

When interpreting the results, the following methodological problems must be taken into account:

– As is usual with surveys, an effect of social desirability which distorts the results in an positive direction cannot be excluded for the two sub-studies. An analysis of the n = 50 clowns who did not complete the questionnaire revealed that these were less likely to be a member of a clown association (a variable which did not influence the outcome estimation, see 3.1.4).

– Originally, a survey of patients was also planned. However, it turned out that there were such large selection effects influencing the practical implementation (approx. 20% return) that the data collected in this manner would not have had any significance.

– In the Hamburg hospital survey, the dependence of clown performances and parental evaluations could not be analysed at multiple levels due to the low number of responses. A group comparison subjected to an analysis of variance showed, however, that parental satisfaction did not depend on the clowns who performed.

Overall, based on the data available at the present time, the use of hospital clowns in paediatric wards can be recommended. In locations where hospital clowns work, interdisciplinary communication and integration as well as respectful and appreciative interaction are essential. Explicit rules of conduct as well as a professional feedback mechanism would help to prevent potential negative side effects. Moreover, it should be checked whether hospital clowning could also be used to reduce fear, anxiety and stress before and during stressful interventions under routine conditions. There is still an enormous amount of research to be done: hospital clowning should be seen as a regular supportive intervention to be studied using standardized methods of evaluation, intervention and health care provision research just like traditional medical measures. Especially the effect of clown visits during intrusive medical procedures seems to be a promising research target. The present study generated health care data on hospital clowning in paediatrics in Germany for the first time, showing that it is easy to implement, is perceived as effective in the short term and thus a useful and practical measure to help suffering children in the hospital system to cope better with their situation.

## Competing interests

This study was conducted with the financial assistance of the registered association “Humor Hilft Heilen”. The authors declare that they have no further competing interests.

## Authors’ contributions

CB was responsible for the conception, study design, calculations and the final manuscript. AKS participated in the study design and the calculations, carried out the collection of data and drafted the manuscript. NW participated in the design, performed additional analyses and revised the manuscript. MSM conceived the study, participated in the coordination and revised the paper. All authors read and approved the final manuscript.

## Pre-publication history

The pre-publication history for this paper can be accessed here:

http://www.biomedcentral.com/1471-2431/13/166/prepub

## Supplementary Material

Additional file 1 Questionnaire for clowns. Click here for file

Additional file 2 Questionnaire for parents. Click here for file

Additional file 3 Questionnaire for clinical stuff. Click here for file
