# Supplementary material for: Clowning as a supportive measure in paediatrics - a survey of clowns, parents and nursing staff
Source: BMC Pediatr. 2013 Oct 10;13:166. doi: 10.1186/1471-2431-13-166 (PMC3851858; doi:10.1186/1471-2431-13-166)
Supplement: Additional file 1 — Questionnaire for clowns. [file 1471-2431-13-166-S1.doc]

| 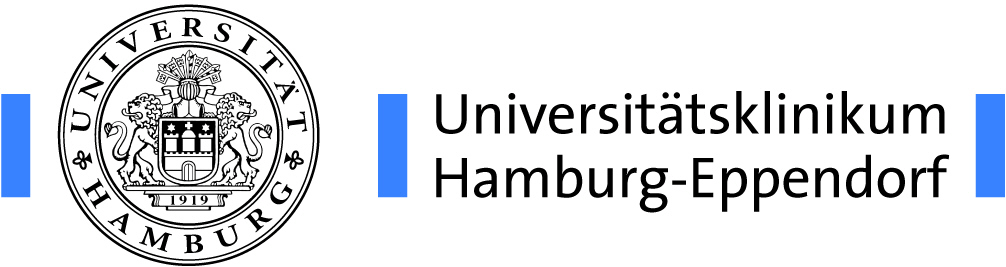 | **Klinik für Kinder- und Jugend­psychiatrie, -psychotherapie**  **und -psychosomatik**  Prof. Dr. med. M. Schulte-Markwort  Direktor |  | **Zentrum für**  **Psychosoziale Medizin**  Martinistraße 52  20246 Hamburg  Telefon: (040) 7410-53697  Telefax: (040) 7410-55105  www.uke.de |
| --- | --- | --- | --- |
|  |  |  |
| An alle Klinikclowns |  | | Jun.-Prof. Claus Barkmann  (Dr. phil. M.P.H. Dipl.-Psych.)  AG Epidemiologie und Evaluation |
|  | | | 29. August 2026/bark |

# Fragebogen zur Clownerie in Kinderkliniken

Sehr geehrte Klinikclownin, sehr geehrter Klinikclown,

wir möchten Sie um Unterstützung bei einer bundesweiten Umfrage bitten, die von unserer Klinik in Kooperation mit der Stiftung „Humor hilft heilen“ und xxxxx durchgeführt wird. Dabei geht es um die Beschreibung Ihrer alltäglichen Arbeit als Klinikclown/in in Kinderkliniken sowie Ihre diesbezüglichen Einstellungen und Erfahrungen. Die Ergebnisse sollen über das Tätigkeitsfeld der Klinikclownerie in Deutschland aufklären und eine systematische Beschreibung der diesbezüglichen Versorgungssituation für klinisch-pädiatrische Patienten ermöglichen. Bitte helfen Sie uns in dieser Sache, indem Sie den beiliegenden Fragebogen beantworten.

- Die Teilnahme an der Umfrage ist freiwillig, eine Nichtteilnahme bleibt natürlich folgenlos.
- Die Erhebung der Daten erfolgt in anonymisierter Form. Es werden also keine persönlichen Angaben wie Namen oder Adresse erfragt. Trotzdem benötigen wir einige andere persönliche Angaben, um zu wissen, wer überhaupt als Klinikclown/in arbeitet.
- Eine Weitergabe der erhobenen Daten an Dritte erfolgt nicht.
- Die Regelungen des geltenden Datenschutzgesetzes werden eingehalten.

In der Anlage finden Sie den Fragebogen und einen Rückumschlag. Bitte füllen Sie diesen Bogen allein, ohne sich abzusprechen und vollständig aus. Den ausgefüllten Fragebogen senden Sie bitte in dem bereits frankierten und adressierten Rückumschlag an uns zurück. Bitte geben Sie keinen Absender an. Über die Ergebnisse werden Sie gegen Ende des Jahres durch xxxx informiert. Falls Sie noch Fragen haben, können Sie uns gern unter der oben genannten Telefonnummer kontaktieren.

Vielen Dank für Ihre Unterstützung!

C. Barkmann K. Siem (Doktorandin)

### 1. Allgemeines zur Tätigkeit als Clown

1. Seit wievielen Jahren arbeiten Sie bereits als Klinikclown? Jahre

2. Wie haben Sie Ihre Tätigkeit als Klinikclown erlernt?

O Clownschule

O Hospitation

O Seminare

O Sonstiges:

3. Was ist Ihre Hauptmotivation für diese Tätigkeit?

4. Auf welcher formalen Basis üben Sie diese Tätigkeit aus?

O Ehrenamtlich

O Selbstständig/Honorarbasis

O Angestellt

O Anderes:

5. Wie hoch ist Ihr durchschnittlicher Stundenlohn in Euro derzeit: €

6. Welche Institution zahlt Ihnen diesen Lohn aus?

O ohne Lohn/ehrenamtlich

O Stiftung

O Verein

O Krankenhaus

O Anderes:

7. An wievielen Fortbildungen bzw. Coachings zur Klinik-
 clownerie haben Sie im letzten Jahr (2010) teilgenommen?

1. Mit welchen Personengruppen arbeiten Sie zu welchem prozentualen Anteil?

O Patienten in pädiatrischen Abteilungen %

O Kinder und Jugendliche in der Psychiatrie %

O Erwachsene in der Psychiatrie %

O Patienten in der geriatrischen Abteilung %

O Senioren in Altenheimen %

O Andere: %

Summe = 100 %

### 2. Rahmenbedingungen der Auftritte in Kinderkliniken

1. Wieviele Stunden pro Woche arbeiten Sie durchschnittlich
   als Klinikclown bei pädiatrischen Patienten?
2. Wieviele Patienten werden von Ihnen pro Woche
   durchschnittlich versorgt?
3. Wie lange dauert ein durchschnittlicher
   Auftritt bei einem Patienten in Minuten?
4. Wieviel Prozent der Patienten sehen Sie im Durchschnitt …

1 mal %

2-4 mal %

5-10 mal %

mehr als 10 mal %

Summe = 100 %

1. Treten Sie auch vor Patientengruppen auf? O nein O ja

Wenn ja, wie häufig pro Woche?

Wieviele Kinder sind in diesen Patientengruppen im Mittel?

1. Ab welchem Alter besuchen Sie Patienten? Jahre
2. Ihre Kerngruppe sind hauptsächlich Patienten von bis Jahre.
3. Wieviele verschiedene Einrichtungen versorgen Sie
   normalerweise gleichzeitig?
4. In welchem Rhythmus besuchen Sie diese Einrichtungen normalerweise?

O 1x wöchentlich

O 14-tägig

O 1x monatlich

O Anderer:

### 3. Ablauf der Auftritte in Kinderkliniken

1. Gehört zu Ihrer Verkleidung ein Arztkittel? O nein O ja
2. Wieviele Stunden in der Woche spielen Sie durchschnittlich
   gemeinsam mit einer Kollegin oder einem Kollegen? Std.
3. Angenommen, man würde dem Clown drei Hauptrollen zuschreiben, welche Rollen nehmen Sie zu wieviel Prozent bei Ihren Auftritten ein (Summe = 100%)?

Rolle 1: Der Clown als dummer August, welcher vorwiegend
Witz und Humor einsetzt. %

Rolle 2: Der Clown als Unterhalter, welcher hauptsächlich
Elemente wie Musik, Zauberei, Pantomime oder Akrobatik nutzt. %

Rolle 3: Der Clown als individueller Fürsorger,
der dem Kind überwiegend seelische Unterstützung bietet
und ihm Gesellschaft leistet. %

1. Zu wieviel Prozent gehören nachfolgende Elemente zu Ihren Auftritten?

O Musik %

O Zauberei %

O Pantomime %

O Akrobatik %

O Sonstiges: %

Summe = 100 %

1. Es gibt auch immer einen Anteil an Patienten, die einen Clownbesuch ablehnen.
   Wieviel Prozent der Patienten, die eigentlich zu ihrere Zielgruppe gehören,
   g, lehnen diesen bei Ihnen ab? %
2. Wer spricht die Ablehnung aus (geschätzt in %)?

O Patient selbst %

O Eltern %

O Pflegekräfte %

O Ärzte %

Summe = 100 %

1. Was sind die Gründe für die Ablehnung (geschätzt in Prozent)?

O keine Zeit %

O keine Lust %

O Angst %

O Schmerzen %

O Ruhebedürftigkeit/Schlaf %

O Sonstiges: %

Summe = 100 %

### 4. Patienten, Eltern und Klinikpersonal

| Was glauben Sie? Welche Prozesse können Sie durch Ihre Auftritte bei den Patienten gezielt fördern? | | | | | | |
| --- | --- | --- | --- | --- | --- | --- |
| Meine Auftritte fördern … | | gar  nicht | etwas | mittel-mäßig | ziem-lich | sehr |
| 1. | das Allgemeinbefinden | 0 | 1 | 2 | 3 | 4 |
| 2. | eine Neubewertung der Situation | 0 | 1 | 2 | 3 | 4 |
| 3. | den Abbau von Ängsten | 0 | 1 | 2 | 3 | 4 |
| 4. | die Fantasie | 0 | 1 | 2 | 3 | 4 |
| 5. | den Heilungsprozess | 0 | 1 | 2 | 3 | 4 |
| 6. | den Stressabbau | 0 | 1 | 2 | 3 | 4 |
| 7. | Anderes:_______________________________ | 0 | 1 | 2 | 3 | 4 |

| Was glauben Sie? Welche Wirkung haben Ihre Auftritte auf die Eltern? | | | | | | |
| --- | --- | --- | --- | --- | --- | --- |
| Meine Auftritte bedeuten für die Eltern eine … | | gar  nicht | etwas | mittel-mäßig | ziem-lich | sehr |
| 1. | Ablenkung | 0 | 1 | 2 | 3 | 4 |
| 2. | Entlastung | 0 | 1 | 2 | 3 | 4 |
| 3. | Ruhestörung | 0 | 1 | 2 | 3 | 4 |
| 4. | Stimmungsaufhellung | 0 | 1 | 2 | 3 | 4 |
| 5. | Überforderung | 0 | 1 | 2 | 3 | 4 |
| 6. | Unannehmlichkeit | 0 | 1 | 2 | 3 | 4 |
| 7. | Unterstützung | 0 | 1 | 2 | 3 | 4 |
| 8. | Anderes:_______________________________ | 0 | 1 | 2 | 3 | 4 |

| Was glauben Sie? Welche Wirkung haben Ihre Auftritte auf das Krankenhauspersonal? | | | | | | |
| --- | --- | --- | --- | --- | --- | --- |
| Meine Auftritte bedeuten für das Krankenhauspersonal eine … | | gar-nicht | etwas | mittel-mäßig | ziem-lich | sehr |
| 1. | Abwechslung | 0 | 1 | 2 | 3 | 4 |
| 2. | Belastung | 0 | 1 | 2 | 3 | 4 |
| 3. | Entlastung | 0 | 1 | 2 | 3 | 4 |
| 4. | Störung der Abläufe | 0 | 1 | 2 | 3 | 4 |
| 5. | Verbesserung der Arbeitsatmosphäre | 0 | 1 | 2 | 3 | 4 |
| 6. | Anderes:_______________________________ | 0 | 1 | 2 | 3 | 4 |

| Was glauben Sie? In welchem Ausmaß wird Ihre Tätigkeit als Klinikclown von folgenden Gruppen wertgeschätzt? | | | | | | |
| --- | --- | --- | --- | --- | --- | --- |
|  | | gar  nicht | etwas | mittel-mäßig | ziem-lich | sehr |
| 1. | Patienten | 0 | 1 | 2 | 3 | 4 |
| 2. | Eltern | 0 | 1 | 2 | 3 | 4 |
| 3. | Pflegepersonal | 0 | 1 | 2 | 3 | 4 |
| 4. | Ärzte | 0 | 1 | 2 | 3 | 4 |

### 5. Arbeitszufriedenheit

|  | | | | | | | | |
| --- | --- | --- | --- | --- | --- | --- | --- | --- |
| Wie zufrieden sind Sie mit ... | | **sehr unzu-**  **frieden** |  |  |  |  |  | **sehr zu-**  **frieden** |
| 1. | den Kollegen, mit denen Sie zusammenarbeiten? | 0 | 0 | 0 | 0 | 0 | 0 | 0 |
| 2. | dem unmittelbar Vorgesetzten, der Ihnen Anweisungen gibt? | 0 | 0 | 0 | 0 | 0 | 0 | 0 |
| 3. | der Tätigkeit inhaltlich? | 0 | 0 | 0 | 0 | 0 | 0 | 0 |
| 4. | den Arbeitsbedingungen (Hilfsmittel, Räume, etc.)? | 0 | 0 | 0 | 0 | 0 | 0 | 0 |
| 5. | der regionalen Vereins-organisation und -leitung | 0 | 0 | 0 | 0 | 0 | 0 | 0 |
| 6. | den persönlichen Entwicklungsmöglichkeiten? | 0 | 0 | 0 | 0 | 0 | 0 | 0 |
| 7. | der Höhe der Bezahlung? | 0 | 0 | 0 | 0 | 0 | 0 | 0 |
| 8. | der Einteilung der Arbeitszeit? | 0 | 0 | 0 | 0 | 0 | 0 | 0 |
| 9. | der Sicherheit des „Arbeitsplatzes“? | 0 | 0 | 0 | 0 | 0 | 0 | 0 |
| 10. | der Tätigkeit **insgesamt**? | 0 | 0 | 0 | 0 | 0 | 0 | 0 |

| Wie zufrieden sind Sie mit den verschiedenen Seiten Ihrer Tätigkeit? | | | | | | |
| --- | --- | --- | --- | --- | --- | --- |
|  | | gar  nicht | etwas | mittel-mäßig | ziem-lich | sehr |
| 1. | Beziehungen zu anderen Berufsgruppen | 0 | 1 | 2 | 3 | 4 |
| 2. | Weiterbildungsmöglichkeiten | 0 | 1 | 2 | 3 | 4 |
| 3. | Bundesweite Vereinsorganisation | 0 | 1 | 2 | 3 | 4 |
| 4. | Kooperation mit Kliniken | 0 | 1 | 2 | 3 | 4 |
| 5. | Allgemeine Anerkennung | 0 | 1 | 2 | 3 | 4 |

Wenn Sie mit einem der genannten Aspekte besonders unzufrieden sind, können Sie hier eintragen, warum:

Was schätzen Sie, wieviele Jahre werden Sie diesen Beruf
voraussichtlich noch ausüben? Jahre

| Allgemeine Fragen zur Arbeits- und Berufszufriedenheit | | | | | | |
| --- | --- | --- | --- | --- | --- | --- |
| Wie sehr stimmen Sie den folgenden Aussagen zu? | | gar  nicht | etwas | mittel-mäßig | ziem-lich | sehr |
| 1. | Ich habe eine wirklich interessante Tätigkeit. | 0 | 1 | 2 | 3 | 4 |
| 2. | Nach getaner Arbeit habe ich doch öfter das Gefühl wirklich etwas geleistet zu haben. | 0 | 1 | 2 | 3 | 4 |
| 3. | Ich glaube, ich habe mehr Spaß an meiner Tätigkeit als andere Leute. | 0 | 1 | 2 | 3 | 4 |
| 4. | Ich glaube, dass ich mit meiner Arbeit zufriedener bin als andere. | 0 | 1 | 2 | 3 | 4 |
| 5. | Wenn ich könnte, würde ich gerne den Beruf als Klinikclown wechseln. | 0 | 1 | 2 | 3 | 4 |
| 6. | Meistens übe ich diese Tätigkeit gerne aus. | 0 | 1 | 2 | 3 | 4 |
| 7. | Das tägliche Betriebseinerlei geht mir oft auf die Nerven. | 0 | 1 | 2 | 3 | 4 |
| 8. | Mit meiner derzeitigen Tätigkeit bin ich ganz zufrieden. | 0 | 1 | 2 | 3 | 4 |
| 9. | Ich habe oft eine Abneigung gegen meine Tätigkeit als Klinikclown. | 0 | 1 | 2 | 3 | 4 |
| 10. | Ich langweile mich oft bei meiner Arbeit als Klinikclown. | 0 | 1 | 2 | 3 | 4 |

### 8. Verschiedenes

| Wie sehr stimmen Sie den folgenden Aussagen zu? | | | | | | |
| --- | --- | --- | --- | --- | --- | --- |
| Stimmt… | | gar  nicht | etwas | mittel-mäßig | ziem-lich | sehr |
| 1. | Im letzten Jahr wurden Klinikclowns verstärkt nachgefragt. | 0 | 1 | 2 | 3 | 4 |
| 2. | Als Klinikclown stehe ich unter psychischem Stress. | 0 | 1 | 2 | 3 | 4 |
| 3. | Wie gut ist das Image von Klinikclowns in der Öffentlichkeit? | 0 | 1 | 2 | 3 | 4 |
| 4. | Die Kooperation mit den Kliniken würde ich allgemein als „sehr gut“ bezeichnen. | 0 | 1 | 2 | 3 | 4 |
| 5. | Als Clown im Krankenhaus fühle ich mich als Störfaktor. | 0 | 1 | 2 | 3 | 4 |
| 6. | Die Patienten profitieren auch noch nach dem Auftritt von dem Erlebnis. | 0 | 1 | 2 | 3 | 4 |
| 7. | Als Clown übe ich auch schon mal unterschwellig Kritik am Krankenhaussystem. | 0 | 1 | 2 | 3 | 4 |
| 8. | Im letzten Jahr wuchs das Interesse der Öffentlichkeit an Klinikclownerie. | 0 | 1 | 2 | 3 | 4 |

### 9. Persönliche Angaben

1. Heutiges Datum:
2. Geschlecht: O männlich O weiblich
3. Alter in Jahren:
4. Geburtsland: O Deutschland O anderes Land:
5. Familienstand: O ledig / ohne festen Partner

O verheiratet / in fester Partnerschaft

O geschieden / getrennt lebend / verwitwet

1. Zahl eigener Kinder:
2. Schulabschluss:

O kein Schulabschluss

O Sonderschule

O Haupt-/Volksschule

O Realschule

O (Fach-)Abitur

O (Fach-)Hochschule/Universität

O anderer:

1. Berufsausbildung:
2. Welche beruflichen Tätigkeiten üben Sie derzeit außer der Klinikclownerie noch aus?

1. Wochenstunden

2. Wochenstunden

3. Wochenstunden

4. Wochenstunden

Gibt es noch etwas, dass Sie uns mitteilen möchten? O nein O ja, nämlich:

**Bitte prüfen Sie Ihre Angaben noch einmal auf Vollständigkeit (nur vollständig ausgefüllte Fragebögen sind verwertbar!) und senden Sie uns dann den Bogen im beiliegenden Rückumschlag zu.**

**Wir bedanken uns herzlich für Ihre Mithilfe!**
