# Supplementary material for: Clowning as a supportive measure in paediatrics - a survey of clowns, parents and nursing staff
Source: BMC Pediatr. 2013 Oct 10;13:166. doi: 10.1186/1471-2431-13-166 (PMC3851858; doi:10.1186/1471-2431-13-166)
Supplement: Additional file 2 — Questionnaire for parents. [file 1471-2431-13-166-S2.doc]

# Fragebogen zur Klinikclownerie für Eltern

### 1. Persönliche Angaben

1. Heutiges Datum:

2. Ausgefüllt von: O Mutter O Vater O andere:

3. Ihr Alter in Jahren:

4. Alter Ihres Kindes in Jahren:

5. Geschlecht Ihres Kindes: O männlich O weiblich

### 3. Zum Krankenhausaufenthalt

1. Art der Station (z. B. Kinderneurologie):

2. Wieviele Tage dauert dieser Krankenhausaufenthalt bereits? Tage

3. Der wievielte Krankenhausaufenthalt von mehr als drei Tagen ist dies für Ihr Kind

insgesamt?

### 3. Clownauftritte

|  | gar  nicht | etwas | mittel-mäßig | ziem-lich | sehr |
| --- | --- | --- | --- | --- | --- |
| 1. Was glauben Sie? Wie gut können Sie die Wirkung  der Klinikclownerie auf Ihr Kind einschätzen? | 0 | 1 | 2 | 3 | 4 |

2. Der wievielte Clown-Besuch war dies für Ihr Kind bei diesem Klinikaufenthalt?

3. Wie häufig wäre Ihrer Meinung nach ein Clownsbesuch bei Ihrem Kind sinnvoll?

O gar nicht

O 1 bis 2mal monatlich

O 1 bis 2mal wöchentlich

O täglich

| Was glauben Sie? Was wurde durch den Clown-Besuch bei Ihrem Kind gezielt gefördert? | | | | | | |
| --- | --- | --- | --- | --- | --- | --- |
| Die Clown-Besuche fördern … | | gar  nicht | etwas | mittel-mäßig | ziem-lich | sehr |
| 1. | eine Stimmungsaufhellung | 0 | 1 | 2 | 3 | 4 |
| 2. | eine Neubewertung der Krankheitssituation | 0 | 1 | 2 | 3 | 4 |
| 3. | den Abbau von Ängsten | 0 | 1 | 2 | 3 | 4 |
| 4. | die Fantasie | 0 | 1 | 2 | 3 | 4 |
| 5. | den Heilungsprozess | 0 | 1 | 2 | 3 | 4 |
| 6. | den Stressabbau | 0 | 1 | 2 | 3 | 4 |
| 7. | Anderes:_______________________________ | 0 | 1 | 2 | 3 | 4 |

| Welche Wirkung hatte der Auftritt auf Sie als Elternteil? | | | | | | |
| --- | --- | --- | --- | --- | --- | --- |
| Der Auftritt bedeutet für mich eine … | | gar  nicht | etwas | mittel-mäßig | ziem-lich | sehr |
| 1. | Ablenkung | 0 | 1 | 2 | 3 | 4 |
| 2. | Entlastung | 0 | 1 | 2 | 3 | 4 |
| 3. | Ruhestörung | 0 | 1 | 2 | 3 | 4 |
| 4. | Stimmungsaufhellung | 0 | 1 | 2 | 3 | 4 |
| 5. | Überforderung | 0 | 1 | 2 | 3 | 4 |
| 6. | Unannehmlichkeit | 0 | 1 | 2 | 3 | 4 |
| 7. | Unterstützung | 0 | 1 | 2 | 3 | 4 |
| 8. | Anderes:_______________________________ | 0 | 1 | 2 | 3 | 4 |

Gibt es etwas, was die Clowns anders oder besser machen könnten? O nein O ja, nämlich:

| Wie sehr stimmen Sie den folgenden Aussagen zu? | | | | | | |
| --- | --- | --- | --- | --- | --- | --- |
| Stimmt… | | gar  nicht | etwas | mittel-mäßig | ziem-lich | sehr |
| 1. | Vor dem ersten Clown-Besuch hatte mein Kind Angst vor dem Clown. | 0 | 1 | 2 | 3 | 4 |
| 2. | Es gibt genug andere Beschäftigungs-möglichkeiten für Kinder auf dieser Station. | 0 | 1 | 2 | 3 | 4 |
| 3. | Ich glaube, dass mein Kind auch noch nach dem Clown-Besuch von dem Erlebnis profitiert. | 0 | 1 | 2 | 3 | 4 |
| 4. | Nach dem Clown-Besuch unterhalten mein Kind und ich uns noch über das Erlebnis. | 0 | 1 | 2 | 3 | 4 |
| 5. | Über einen erneuten Clown-Besuch würde mein Kind sich freuen. | 0 | 1 | 2 | 3 | 4 |
| 6. | Clown-Besuche sind auch für erwachsene Patienten sinnvoll. | 0 | 1 | 2 | 3 | 4 |
| 7. | Die Anwesenheit eines Clowns würde meinem Kind während spezieller Interventionen (z. B. Blutabnahme) helfen. | 0 | 1 | 2 | 3 | 4 |
| 8. | Insgesamt bin ich mit den Clown-Besuchen zufrieden. | 0 | 1 | 2 | 3 | 4 |

**Vielen Dank für die Mitarbeit!**
