# Supplementary material for: Clowning as a supportive measure in paediatrics - a survey of clowns, parents and nursing staff
Source: BMC Pediatr. 2013 Oct 10;13:166. doi: 10.1186/1471-2431-13-166 (PMC3851858; doi:10.1186/1471-2431-13-166)
Supplement: Additional file 3 — Questionnaire for clinical stuff. [file 1471-2431-13-166-S3.doc]

# Fragebogen zur Klinikclownerie für das Pflegepersonal

### 1. Persönliche Angaben

1. Heutiges Datum:

2. Geschlecht: O männlich O weiblich

3. Alter in Jahren:

4. Berufsausbildung:

5. Art der Beschäftigung: O Vollzeit O Teilzeit

### 2. Wirkung auf Patienten, Eltern und Klinikpersonal

|  | gar  nicht | etwas | mittel-mäßig | ziem-lich | sehr |
| --- | --- | --- | --- | --- | --- |
| Was glauben Sie? Wie gut können Sie die Wirkung der Klinikclownerie auf Ihre Patienten einschätzen? | 0 | 1 | 2 | 3 | 4 |

| Was wird Ihrer Meinung nach durch die Clown-Besuche bei den Patienten gezielt gefördert? | | | | | | |
| --- | --- | --- | --- | --- | --- | --- |
| Die Clown-Besuche fördern bei den Patienten … | | gar  nicht | etwas | mittel-mäßig | ziem-lich | sehr |
| 1. | eine Stimmungsaufhellung | 0 | 1 | 2 | 3 | 4 |
| 2. | eine Neubewertung der Krankheitssituation | 0 | 1 | 2 | 3 | 4 |
| 3. | den Abbau von Ängsten | 0 | 1 | 2 | 3 | 4 |
| 4. | die Fantasie | 0 | 1 | 2 | 3 | 4 |
| 5. | den Heilungsprozess | 0 | 1 | 2 | 3 | 4 |
| 6. | den Stressabbau | 0 | 1 | 2 | 3 | 4 |
| 7. | Anderes:_______________________________ | 0 | 1 | 2 | 3 | 4 |

| Welche Wirkung haben die Clown-Besuche Ihrer Meinung nach auf die Eltern? | | | | | | |
| --- | --- | --- | --- | --- | --- | --- |
| Die Auftritte bedeuten für die Eltern eine … | | gar  nicht | etwas | mittel-mäßig | ziem-lich | sehr |
| 1. | Ablenkung | 0 | 1 | 2 | 3 | 4 |
| 2. | Entlastung | 0 | 1 | 2 | 3 | 4 |
| 3. | Ruhestörung | 0 | 1 | 2 | 3 | 4 |
| 4. | Stimmungsaufhellung | 0 | 1 | 2 | 3 | 4 |
| 5. | Überforderung | 0 | 1 | 2 | 3 | 4 |
| 6. | Unannehmlichkeit | 0 | 1 | 2 | 3 | 4 |
| 7. | Unterstützung | 0 | 1 | 2 | 3 | 4 |
| 8. | Anderes:_______________________________ | 0 | 1 | 2 | 3 | 4 |

| Welche Wirkung haben die Clown-Besuche auf Sie selbst? | | | | | | |
| --- | --- | --- | --- | --- | --- | --- |
| Die Clown-Besuche bedeuten für mich eine … | | gar-nicht | etwas | mittel-mäßig | ziem-lich | sehr |
| 1. | Abwechslung | 0 | 1 | 2 | 3 | 4 |
| 2. | Belastung | 0 | 1 | 2 | 3 | 4 |
| 3. | Entlastung | 0 | 1 | 2 | 3 | 4 |
| 4. | Störung der Abläufe | 0 | 1 | 2 | 3 | 4 |
| 5. | Verbesserung der Arbeitsatmosphäre | 0 | 1 | 2 | 3 | 4 |
| 6. | Anderes:_______________________________ | 0 | 1 | 2 | 3 | 4 |

|  | | | | | | |
| --- | --- | --- | --- | --- | --- | --- |
| Was glauben Sie: In welchem Ausmaß wird die Tätigkeit als Klinikclown von folgenden Gruppen wertgeschätzt? | | gar  nicht | etwas | mittel-mäßig | ziem-lich | sehr |
| 1. | Patienten | 0 | 1 | 2 | 3 | 4 |
| 2. | Eltern | 0 | 1 | 2 | 3 | 4 |
| 3. | Pflegepersonal | 0 | 1 | 2 | 3 | 4 |
| 4. | Ärzte | 0 | 1 | 2 | 3 | 4 |

### 3. Letzte Fragen

| Wie sehr stimmen Sie den folgenden Aussagen zu? | | | | | | |
| --- | --- | --- | --- | --- | --- | --- |
| Stimmt… | | gar  nicht | etwas | mittel-mäßig | ziem-lich | sehr |
| 1. | Ich wünsche mir mehr Clown-Besuche auf dieser Station. | 0 | 1 | 2 | 3 | 4 |
| 2. | Ich wünsche mir weniger Clown-Besuche auf dieser Station. | 0 | 1 | 2 | 3 | 4 |
| 3. | Die Clowns sind eine Bereicherung für den Stationsalltag. | 0 | 1 | 2 | 3 | 4 |
| 4. | Die Clowns sind ein Gesprächsthema zwischen mir und den Patienten. | 0 | 1 | 2 | 3 | 4 |
| 5. | Klinikclowns üben unterschwellig Kritik am System „Krankenhaus“. | 0 | 1 | 2 | 3 | 4 |
| 6. | Ich hole Clowns zu speziellen Interventionen (z.B. Blutabnahme) hinzu. | 0 | 1 | 2 | 3 | 4 |
| 7. | Die Clowns erfahren von den Kindern mehr Sympathien als das Pflegepersonal. | 0 | 1 | 2 | 3 | 4 |

Gibt es etwas, was man an den Clown-Besuchen verbessern könnte? O nein O ja, nämlich:

**Vielen Dank für die Mitarbeit!**
